# Pathological Insights Into the Relationship Between Medial and Intimal Calcification in Lower Extremity Artery Disease

**DOI:** 10.1016/j.jacadv.2026.102882

**Published:** 2026-06-11

**Authors:** Tsukasa Kato, Sho Torii, Manabu Shiozaki, Norihito Nakamura, Kazuki Aihara, Yu Sato, Yuki Matsumoto, Yuta Terabe, Osamu Iida, Takahiro Tokuda, Tomohiro Iwata, Hikaru Onishi, Kaho Hashimoto, Ryosuke Omura, Daiki Suzuki, Yuto Ono, Ken Miura, Hiroyuki Watanabe, Yuji Ikari, Gaku Nakazawa

**Affiliations:** a Department of Cardiology, Akita University School of Medicine, Akita, Japan; b Department of Cardiology, Tokai University School of Medicine, Isehara, Japan; c Limb Salvage Center, Kasukabe Chuo General Hospital, Kasukabe, Japan; d Cardiovascular Division, Osaka Keisatsu Hospital, Osaka, Japan; e Department of Cardiology, Nagoya Heart Center, Nagoya, Japan; f Department of Cardiology, Kindai University Faculty of Medicine, Osaka, Japan

**Keywords:** bone formation, chronic limb-threatening ischemia, intimal artery calcification, lower extremity artery disease, medial artery calcification

## Abstract

**Background:**

Medial artery calcification (MAC) is a hallmark of lower extremity artery disease, yet its precise distribution and relationship with intimal artery calcification (IAC) remain poorly understood because current clinical imaging modalities have limited ability to reliably distinguish between the 2.

**Objectives:**

The objectives of the study were to comprehensively characterize the histopathological distribution and morphology of MAC and to determine its spatial relationship with intimal atherosclerotic plaque.

**Methods:**

We performed a systematic, section-by-section histopathological analysis of 3,566 cross-sections from 125 arterial segments in 58 patients with lower extremity artery disease. Calcification was quantified by circumferential arc, and plaque morphology was classified according to the modified American Heart Association criteria.

**Results:**

MAC was highly prevalent (91.2% of segments) and significantly more extensive in below-the-knee than above-the-knee arteries, whereas IAC demonstrated the opposite distribution. Medial bone formation was identified in 16.0% of segments and was strongly associated with the greatest MAC burden, representing an advanced stage of the disease. In a section-level analysis (n = 3,003), the arcs of MAC and IAC were inversely correlated (r = −0.47; *P* < 0.0001) and showed minimal circumferential overlap, indicating distinct spatial segregation. Sections with extensive MAC were primarily associated with nonatherosclerotic intima, whereas advanced intimal plaques occurred predominantly in sections with minimal MAC.

**Conclusions:**

MAC and IAC represent spatially distinct calcific pathologies with an inverse quantitative relationship. Advanced MAC may be associated with reduced development of intimal atherosclerotic plaque and may partly explain the limited response of below-the-knee lesions to conventional endovascular therapies.

Medial artery calcification (MAC) is a systemic vascular disorder distinct from intimal atherosclerosis.^1^ Although rare in the coronary circulation,^2^^,^^3^ MAC is a hallmark of lower extremity artery disease (LEAD), particularly among patients with diabetes mellitus and chronic kidney disease (CKD).^1^ Clinically, MAC is not merely a passive manifestation of aging but is strongly linked to adverse limb outcomes, including major amputation^4^ and poor patency after revascularization.^5^^,^^6^ Despite its profound clinical relevance, the specific contribution of MAC to limb ischemia remains insufficiently understood, in part because traditional vascular paradigms have focused predominantly on intimal atherosclerosis.

A major obstacle to advancing this field is the limited ability of current imaging modalities to differentiate calcification phenotypes. Radiography,^7^ computed tomography,^8^ and intravascular imaging^5^^,^^9^ can detect calcium but have limited ability to reliably distinguish medial from intimal artery calcification (IAC), particularly when both forms coexist. As a result, the true prevalence, distribution, and severity of MAC have likely been underestimated. Moreover, because MAC and IAC frequently overlap in their radiographic appearance, it remains unclear whether they represent synergistic processes contributing to luminal narrowing or distinct pathological entities with fundamentally different spatial patterns.

Histopathology remains the gold standard for differentiating MAC from IAC. However, prior pathological studies have been constrained by modest sample sizes, evaluation limited to the most stenotic segments, or insufficient characterization of intimal plaque morphology.^10^, ^11^, ^12^, ^13^ Although our previous work identified a strong association between hemodialysis and medial calcification, a comprehensive, high-resolution mapping of calcification across the entire lower extremity arterial tree has not been performed.^14^ Understanding the precise interplay between medial and intimal pathology is essential to explain why below-the-knee (BTK) lesions often remain challenging to treat with established endovascular therapies, such as plain balloon angioplasty^15^ and drug-coated balloons.^16^

To address these gaps, we conducted a systematic section-by-section histopathological study of lower extremity arterial segments from patients with LEAD. The objectives of this investigation were: 1) to define, on a section-by-section basis, the distribution and severity of MAC and IAC from the femoral to the BTK arteries; and 2) to elucidate the spatial and quantitative relationship between MAC and intimal atherosclerotic plaque. This study provides detailed characterization of lower extremity arterial calcification and offers new insights into the distinct biology underlying limb vascular disease.

## Methods

### Study population and patient characteristics

This study analyzed arterial specimens obtained from amputation and autopsy cases enrolled in the CV Hills Pathology Registry of LEAD Patients, a multicenter registry involving 15 institutions in Japan. Between November 2019 and September 2025, we enrolled 58 patients (58 limbs) with symptomatic LEAD, defined as Rutherford category 3 to 6. The cohort consisted of 52 patients (89.7%) who underwent major amputation for chronic limb-threatening ischemia (CLTI) or acute limb ischemia, and 6 autopsy cases (10.3%). All autopsy cases had a documented history of LEAD, and only lower extremity arterial specimens were collected and analyzed from these patients. Detailed information on prior revascularization procedures, including modality (surgical, endovascular, or hybrid), target vessel, lesion length, and devices used, was collected when available. Segments with prior stent implantation or surgical grafting were excluded from the histopathological analysis to avoid direct procedural artifacts. The study protocol was approved by the Tokai University Institutional Review Board (No. 19R289), and informed consent was obtained in accordance with the Declaration of Helsinki.

### Tissue processing and histological analysis

Arterial segments were harvested from amputated or autopsy limbs and classified according to anatomical landmarks and radiographic findings. Above-the-knee (ATK) arteries included the superficial femoral artery and popliteal artery, whereas BTK arteries included the tibioperoneal trunk, anterior tibial artery, posterior tibial artery, and peroneal artery. Segments containing stents or surgical grafts were excluded. Because arterial specimens were obtained from amputated limbs or autopsy cases, the number and anatomical distribution of harvested segments were not uniform across patients.

Arterial segments were fixed in 10% formalin, decalcified as required, and serially sectioned at 3 to 4 mm intervals. Sections were stained with hematoxylin and eosin for morphological assessment and Movat Pentachrome stain to delineate the elastic laminae, enabling precise distinction between medial and intimal layers. Digital images were acquired and analyzed using ZEN software (ZEISS). All tissue processing and histological procedures were performed in accordance with previously described protocols.^14^^,^^17^

### Assessment and quantification of calcification

Histological calcifications were classified according to the degree and size of calcium deposits, as described in previous reports.^1^^,^^18^ Microcalcification was defined as calcium deposits measuring ≥0.5 μm but <15 μm; punctate calcification as deposits >15 μm but <1 mm; fragment calcification as deposits ≥1 mm; sheet calcification as calcium involving >90° of the vessel circumference; and nodular calcification as fractured calcific structures that did not extend into the arterial lumen. Medial bone formation was defined by the presence of mature bone tissue within the medial layer, characterized by trabecular structures with marrow-like spaces, clearly distinct from amorphous mineral deposition.

**Figure 1:**
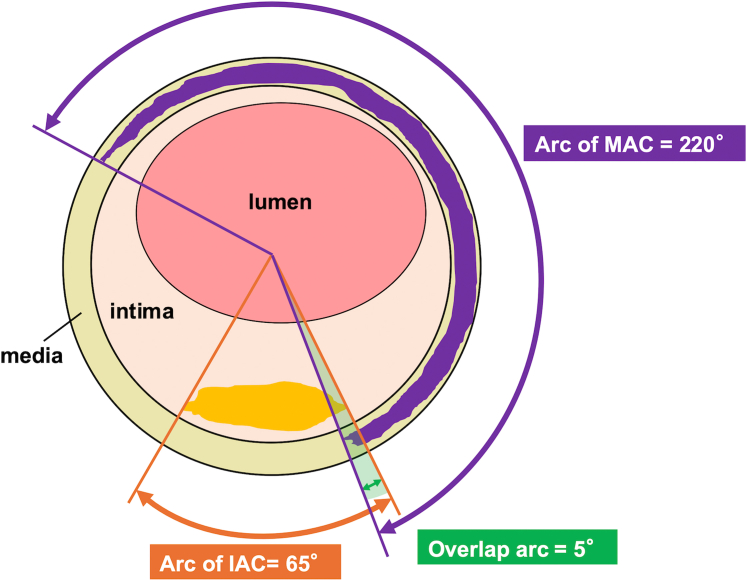
Methods for Measuring Calcification Representative schematic illustration showing the circumferential arcs of MAC, IAC, and their overlap. The arcs of MAC and IAC are measured independently, and the overlap arc represents the circumferential region where both forms of calcification coexist on the same plane of the arterial wall. IAC = intimal artery calcification; MAC = medial artery calcification.

Quantitative measurements were performed to determine the circumferential arc of calcification (0°-360°) in the media and intima independently. Severe calcification was defined as an arc ≥180°. To evaluate the spatial relationship between the 2 calcification types, the *overlap arc* was defined as the circumferential angle in which medial and intimal calcifications coexisted along the same radial vector of the arterial wall (Figure 1).

### Classification of atherosclerotic plaque

Intimal plaque morphology was classified according to the modified American Heart Association criteria.^19^^,^^20^ Adaptive intimal thickening was defined as the natural accumulation of vascular smooth muscle cells within a proteoglycan-rich collagen matrix without lipid deposition or macrophage foam cells. Pathological intimal thickening was defined as a lipid pool composed of apoptotic vascular smooth muscle cells. Fibroatheroma was characterized by the presence of macrophages surrounding the necrotic core with a dense fibrous cap. Thin-cap fibroatheroma was defined as a necrotic core covered by a thin fibrous cap, whereas plaque rupture was identified by a disrupted cap overlying the necrotic core. Fibrous plaque was characterized by collagen-rich neointimal tissue with few vascular smooth muscle cells and without a lipid pool or necrotic core. Fibrocalcific plaque was defined as neointimal proliferation with calcification. Calcified nodule was defined as a calcific lesion protruding into the arterial lumen with an overlying thrombus.

Lesions were grouped as nonatherosclerotic (adaptive intimal thickening, pathological intimal thickening, and fibrous plaque) or atherosclerotic (fibroatheroma, thin-cap fibroatheroma, plaque rupture, fibrocalcific plaque, and calcified nodule) for analytical purposes. Chronic total occlusion was defined as the presence of an organized thrombus completely occluding the arterial lumen.

### Statistical analysis

Continuous variables were expressed as mean ± SD or median (IQR), and categorical variables as counts (percentages). For arterial segment–level comparisons, non-normally distributed continuous variables were compared using the Kruskal-Wallis test when appropriate, and categorical variables were compared using the chi-square test or Fisher exact test, as appropriate. Because multiple histological sections were obtained from the same patients, generalized estimating equations (GEE) were used to account for within-patient clustering in section-level analyses. Patient identification was specified as the subject variable, and an exchangeable working correlation structure with robust sandwich SEs was used. For continuous section-level outcomes, GEE models with a normal distribution and identity link function were used to estimate marginal mean differences. For binary section-level outcomes, including the presence of IAC, GEE models with a binomial distribution and logit link function were used to estimate odds ratios. The exchangeable working correlation structure was selected a priori because multiple sections from the same patient were expected to be correlated, without assuming a specific ordering of sections.

Model assumptions were assessed by reviewing the distributions of outcome variables, fitted values, and residual patterns. Because the relationship between MAC and IAC arcs appeared nonlinear on visual inspection of the scatter plot, the association was further evaluated using Spearman rank correlation and a GEE logistic model in which IAC presence was treated as a binary outcome and MAC arc was modeled per 100° increase. Model-based results are reported as estimates or ORs with 95% CI and *P* values. A 2-sided *P* value <0.01 was considered statistically significant. All statistical analyses were performed using JMP (Student Edition 18.2.1; SAS Institute), GraphPad Prism 7.03, and SPSS (version 27; IBM Corp).

## Results

### Patient and vessel characteristics

A total of 58 patients were included in this study, representing a high-risk LEAD population. The median age was 74 years, and 56.1% were men. Notably, CLTI with Rutherford category ≥ IV was present in 98.2% of patients, and 58.6% were receiving hemodialysis, indicating that the cohort predominantly consisted of individuals with advanced limb ischemia and severe metabolic comorbidities. Most patients had traditional cardiovascular risk factors, including hypertension, diabetes mellitus, dyslipidemia, and CKD. Fifty-two patients (89.7%) had undergone prior revascularization. Detailed procedural characteristics of prior revascularization were not consistently available.

In total, 3,566 histological sections from 125 arterial segments were evaluated, representing a comprehensive section-by-section pathological analysis of lower extremity arteries. Of these, 39 segments (31.2%) were classified as ATK (14 superficial femoral artery and 25 popliteal artery), and 86 segments (68.8%) as BTK (5 tibioperoneal trunk, 35 anterior tibial artery, 27 posterior tibial artery, and 19 peroneal artery). The distribution of arterial segments was not uniform, with a greater proportion of BTK segments reflecting the clinical predominance of distal disease in this high-risk LEAD cohort. The median degree of maximal luminal stenosis was 90% (IQR: 59.5% to 99.5%), and chronic total occlusion was identified in 33 segments (26.4%). When stratified by anatomical level, maximal degree of luminal stenosis did not differ significantly between ATK and BTK segments. In contrast, nonatherosclerotic intima was more frequently observed in BTK segments, whereas atherosclerotic intima predominated in ATK segments. Medial bone formation was also more frequently identified in BTK segments, consistent with the greater burden of medial calcification in this region. Detailed patient and vessel characteristics are summarized in Tables 1 and 2.

**Table 1:** Patient Characteristics (N = 158)

Age (y)	74 (65-79)
Male, n (%)	32 (56.1)
Body mass index (kg/m^2^)	20.9 (17.9-23.6)
Body surface area (m^2^)	1.5 (1.4-1.6)
Hypertension, n (%)	42 (72.4)
Diabetes, n (%)	33 (56.9)
Dyslipidemia, n (%)	24 (41.4)
Smoking, n (%)	17 (32.1)
Coronary artery disease, n (%)	30 (52.6)
Atrial fibrillation, n (%)	5 (8.7)
Chronic kidney disease, n (%)	39 (67.2)
Hemodialysis, n (%)	34 (58.6)
Acute limb ischemia, n (%)	7 (12.1)
Rutherford classification	
≥Ⅳ, n (%)	56 (98.2)
Amputation or autopsy	
Amputation, n (%)	52 (89.7)
Autopsy, n (%)	6 (10.3)
Laboratory data	
Ca (mg/dL)	8.5 (8.2-9.3)
P (mg/dL)	4.0 (3.2-5.0)
PTH-INTACT (pg/mL)	123.5 (77.3-151.5)
TG (mg/dL)	90 (71.5-127.0)
HDL-C (mg/dL)	44.0 (33.3-56.0)
LDL-C (mg/dL)	71.0 (50.0-98.0)
HbA1c (%)	6.2 (5.6-6.9)
Medication	
Antiplatelet use, n (%)	51 (89.4)
Anticoagulant use, n (%)	13 (22.8)
Statin use, n (%)	24 (42.1)

Values are median (IQR) or n (%).

HDL-C = high-density lipoprotein cholesterol; LDL-C = low-density lipoprotein cholesterol; HbA1c = glycosylated hemoglobin; PTH-INTACT = intact parathyroid hormone; TG = triglycerides.

**Table 2:** Vessel Characteristics (N = 125)

		*P* Value
Lesion distribution		
Above the knee, n (%)	39 (31.2)	
Superficial femoral artery, n (%)	14 (11.2)	
Popliteal artery, n (%)	25 (20)	
Below the knee, n (%)	86 (68.8)	
Tibial peroneal trunk, n (%)	5 (4.0)	
Anterior tibial artery, n (%)	35 (28.0)	
Posterior tibial artery, n (%)	27 (21.6)	
Peroneal artery, n (%)	19 (15.2)	
Maximum degree of luminal stenosis	90 (59.5-99.5)	
Above the knee	75 (71.0-90.0)	0.031
Below the knee	90 (53.6-100)	
Prevalence of nonatherosclerotic intima	80 (39.6-97.8)	
Above the knee	44.4 (14.3-77.5)	<0.0001
Below the knee	88.9 (65.2-100)	
Prevalence of atherosclerotic intima	20 (2.2-60.4)	
Above the knee	55.5 (22.5-85.7)	<0.0001
Below the knee	11.1 (0-34.8)	
Chronic total occlusion, n (%)	33 (26.4)	
Above the knee, n (%)	7 (5.6)	0.149
Below the knee, n (%)	26 (20.8)	
Prevalence of intimal calcification	7.1 (0-45.7)	
Above the knee	45.5 (16.7-85.7)	<0.0001
Below the knee	2.8 (0-12.1)	
Arc of intimal calcification	5.5 (0-47.8)	
Above the knee	40.8 (6.7-75.2)	<0.0001
Below the knee	1.2 (0-14.3)	
Prevalence of medial calcification	96.3 (50.0-100)	
Above the knee	63.6 (6.7-100)	0.0002
Below the knee	100 (73.9-100)	
Arc of medial calcification	119.5 (20.0-284.5)	
Above the knee	28.3 (0.8-207.3)	<0.0001
Below the knee	239.5 (53.7-330.3)	
Intimal bone formation, n (%)	24 (19.2)	
Above the knee, n (%)	16 (12.8)	<0.0001
Below the knee, n (%)	8 (6.4)	
Medial bone formation, n (%)	20 (16.0)	
Above the knee, n (%)	3 (2.4)	0.088
Below the knee, n (%)	17 (13.6)	

Values are median (IQR) or n (%).

### Distribution and extent of medial and intimal calcification

**Figure 2:**
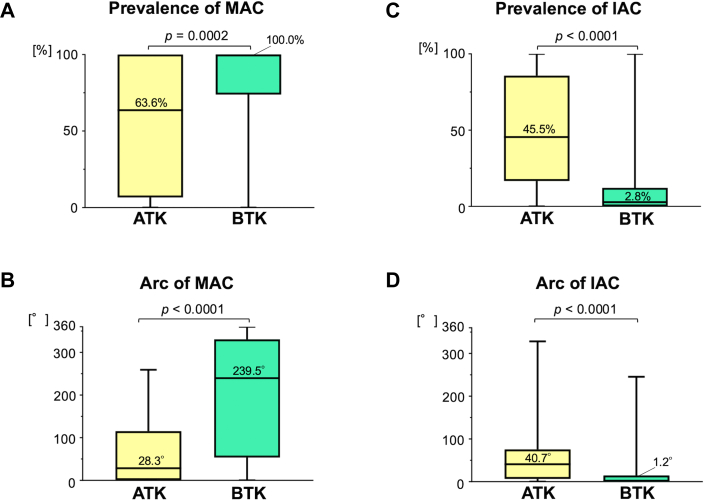
Distribution and Extent of Medial and Intimal Calcification in Above-the-Knee and Below-the-Knee Arterial Segments (A) The prevalence and (B) the arc of MAC were markedly higher in BTK than ATK arterial segments. (C) The prevalence and (D) the arc of IAC were higher in ATK than BTK arterial segments. ATK = above-the-knee; BTK = below-the-knee; other abbreviations as in Figure 1.

MAC was highly prevalent, observed in 114 of 125 arterial segments (91.2%) and in 54 of 58 patients (93.1%). Its distribution showed a clear anatomical gradient: both the prevalence (100.0% [IQR: 73.9% to 100.0%] vs 63.6% [IQR: 6.7% to 100.0%]) and the arc of MAC (239.5° [IQR: 53.7°–330.3°] vs 28.3° [IQR: 0.8°–115.1°]) were markedly greater in BTK than in ATK arteries (*P* = 0.0002 and *P* < 0.0001, respectively) (Figures 2A and 2B). Within each anatomical level, no significant differences were observed among individual BTK segments (tibioperoneal trunk, anterior tibial artery, posterior tibial artery, and peroneal artery) or among ATK segments (superficial femoral artery and popliteal artery) (Table 3). In contrast, IAC demonstrated the opposite pattern. Its prevalence (45.5% [IQR: 16.7% to 85.7%] vs 2.8% [IQR: 0% to 12.1%]) and arc (40.7° [IQR: 6.7°–75.2°] vs 1.2° [IQR: 0°–14.3°]) were significantly greater in ATK than BTK arteries (both *P* < 0.0001) (Figures 2C and 2D). Similar to MAC, no significant segment-level differences were observed within either ATK or BTK arteries. Notably, tibioperoneal trunk segments exhibited a pattern more similar to ATK arteries with respect to both MAC and IAC (Table 3).

**Table 3:** Prevalence and Arc of Calcification by Arterial Segments

Prevalence of Medial Calcification	Median (IQR)	*P* Value
Superficial femoral artery	62.7 (24.0-98.0)	0.74
Popliteal artery	66.7 (0-100.0)	
Anterior tibial artery	97.5 (66.7-100.0)	0.69
Tibial peroneal trunk	90.9 (50.0-100.0)	
Posterior tibial artery	100.0 (89.7-100.0)	
Peroneal artery	100.0 (78.6-100.0)	
Arc of medial calcification		
Superficial femoral artery	25.4 (7.9-97.7)	0.96
Popliteal artery	28.3 (0-124.3)	
Anterior tibial artery	239.0 (57.7-327.8)	0.1
Tibial peroneal trunk	40.0 (8.9-107.3)	
Posterior tibial artery	280.3 (60.7-343.1)	
Peroneal artery	225.7 (37.1-334.9)	
Prevalence of intimal calcification		
Superficial femoral artery	35.6 (11.3-55.6)	0.079
Popliteal artery	55.6 (20.5-92.2)	
Anterior tibial artery	5.6 (0-11.8)	0.077
Tibial peroneal trunk	71.4 (31.8-91.7)	
Posterior tibial artery	2.2 (0-11.4)	
Peroneal artery	0 (0-8.3)	
Arc of intimal calcification		
Superficial femoral artery	26.6 (4.5-175.1)	0.35
Popliteal artery	44.3 (12.3-79.3)	
Anterior tibial artery	2.2 (0-13.3)	0.12
Tibial peroneal trunk	88.5 (24.1-111.9)	
Posterior tibial artery	1.1 (0-15.3)	
Peroneal artery	0 (0-5.6)	

Values are median (IQR).

### Morphology and severity of medial and intimal calcification

MAC demonstrated a clear anatomical distinction in its morphological patterns. BTK sections were dominated by advanced MAC phenotypes, particularly sheet and nodular calcification, whereas ATK sections more frequently exhibited early-stage patterns, such as punctate and fragment calcification. Severe MAC (arc ≥180°) was also more common in BTK (68.3%) than in ATK (27.3%) sections.

**Figure 3:**
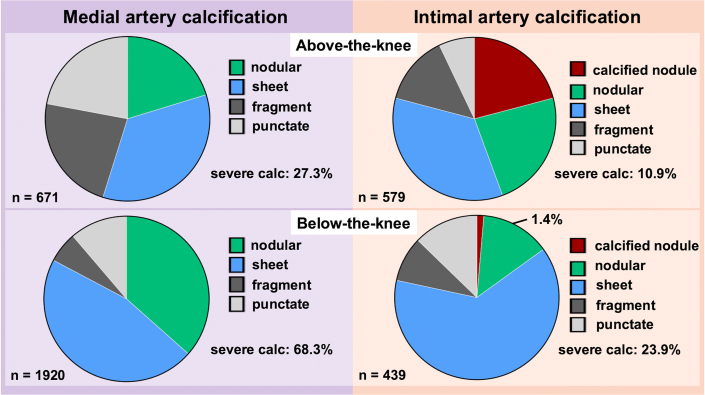
Differences in Morphological Features of Medial and Intimal Calcification in Above-the-Knee and Below-the-Knee Arterial Sections Regarding MAC, advanced calcification patterns (sheet and nodular) predominated in BTK sections, whereas ATK sections showed a comparatively greater presence of early-stage patterns (punctate and fragment). For IAC, calcified nodule was observed almost exclusively in ATK sections.

In contrast, IAC showed a different morphological profile. Calcified nodules were observed almost exclusively in ATK sections, whereas sheet calcification accounted for most IAC lesions in BTK sections. Across all arterial levels, severe IAC was substantially less frequent than severe MAC (Figure 3).

### Medial bone formation and its association with medial calcification severity

**Figure 4:**
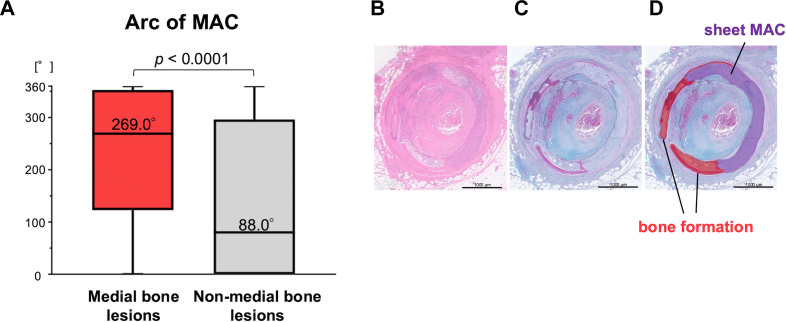
Association Between Medial Bone Formation and Severity of Medial Artery Calcification (A) The presence of medial bone formation was associated with a larger arc of MAC (*P* < 0.0001). Representative histologic images (B, H&E; C, Movat) and the corresponding schematic illustration (D) showing medial bone formation with severe MAC, characterized by trabecular bone structures with marrow-like spaces consistent with mature ossification within the medial layer. Abbreviation as in Figure 1.

Medial bone formation was identified in 20 arterial segments (16.0%) and 101 sections (2.8%). Its presence was strongly associated with markedly greater MAC burden, with a median arc of 269.0° (IQR: 123.0°–352.5°) compared with 88.0° (IQR: 0°–300.0°) in sections without bone formation (*P* < 0.0001). These findings indicate that bone formation represents an advanced stage within the spectrum of MAC progression (Figure 4).

### Medial calcification and intimal plaque phenotype

**Figure 5:**
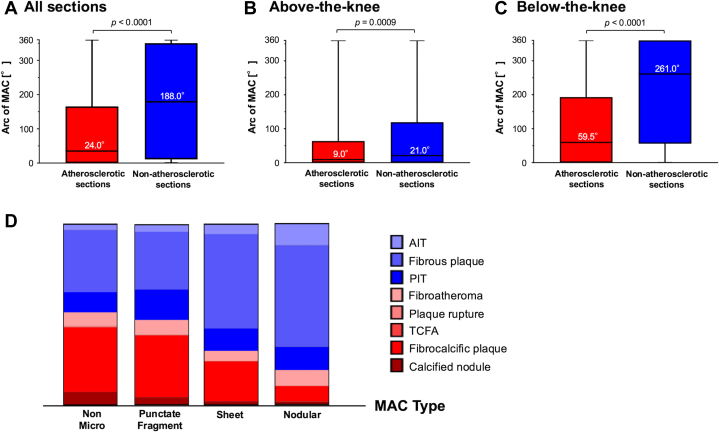
Relationship Between Severity of Medial Artery Calcification and Intimal Plaque Phenotype (A) The arc of MAC was significantly greater in sections with nonatherosclerotic intima than in those with atherosclerotic plaques (*P* < 0.0001). This association remained significant when analyzed separately in (B) ATK and (C) BTK arteries. (D) Intimal plaque phenotypes according to the morphological patterns of MAC. Medial sections exhibiting advanced calcification patterns (sheet or nodular) were most frequently associated with fibrous plaque, whereas sections with early calcification patterns (micro, punctate, or fragment) tended to co-occur with advanced intimal plaques. AIT = adaptive intimal thickening; PIT = pathological intimal thickening; TCFA = thin-cap fibroatheroma; other abbreviation as in Figure 1.

The extent of MAC differed markedly according to intimal plaque phenotype. Sections with nonatherosclerotic intima exhibited substantially larger arcs of MAC compared with those containing atherosclerotic plaques (188.0° [IQR: 17.3°–360.0°] vs 24.0° [IQR: 0°–108.0°]; *P* < 0.0001) (Figure 5A). This pattern remained significant in both ATK and BTK arteries when analyzed separately (Figures 5B and 5C), with a more pronounced difference observed in BTK arteries.

Moreover, advanced MAC patterns—sheet and nodular calcification—were most commonly associated with fibrous plaques, whereas early-stage MAC patterns (micro-, punctate-, and fragment-type calcification) tended to co-occur with advanced intimal plaques, including fibrocalcific plaque and calcified nodules (Figure 5D).

These findings suggest an inverse relationship between MAC severity and intimal plaque advancement, in which regions with extensive MAC are less likely to harbor advanced atherosclerosis, whereas advanced plaques preferentially arise in areas with only minimal MAC.

### Relationship between medial and intimal calcification

**Figure 6:**
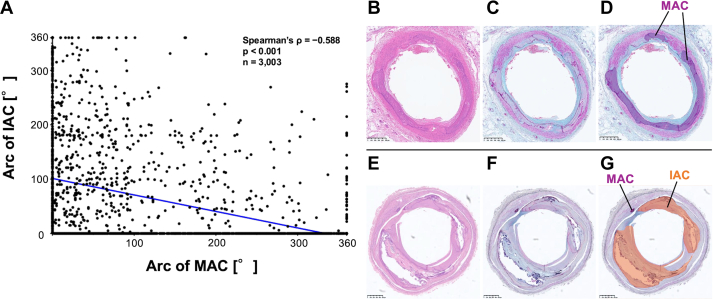
Inverse Association Between Medial and Intimal Calcification Arcs (A) Scatter plot demonstrating an inverse association between the arcs of MAC and IAC. Spearman rank correlation confirmed this inverse association (ρ = −0.588; *P* < 0.001; n = 3,003). A GEE logistic model accounting for within-patient clustering also showed that each 100° increase in MAC arc was associated with lower odds of IAC presence. Representative histologic images (B and E, H&E; C and F, Movat) and the corresponding schematic illustrations (D and G) showing arterial sections in which medial and intimal calcification do not coexist along the same circumferential location. Abbreviations as in Figure 1.

Across all 3,003 sections in which either MAC or IAC was present, the extent of MAC was inversely associated with the extent of IAC. Pearson correlation coefficient, used as a descriptive measure, showed an inverse relationship between MAC and IAC arcs (r = −0.47; 95% CI: −0.50 to −0.44; *P* < 0.0001) (Figure 6A). Because the scatter plot suggested a nonlinear relationship, we additionally performed Spearman rank correlation analysis, which confirmed the inverse association between MAC and IAC arcs (Spearman ρ = −0.588; *P* < 0.001; n = 3,003). In a GEE logistic model accounting for within-patient clustering, each 100° increase in MAC arc was associated with lower odds of IAC presence (OR: 0.39; 95% CI: 0.29-0.53; *P* < 0.001). These findings support a local inverse relationship between MAC burden and the presence of IAC. Representative examples illustrating this pattern are shown in Figures 6B and 6C, where sections dominated by MAC exhibited little or no IAC, and vice versa. These images serve as visual demonstrations of the inverse relationship captured quantitatively in the scatter plot.

**Figure 7:**
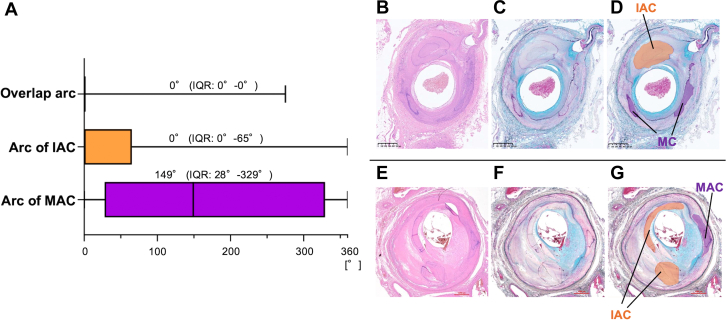
Overlap Arc of Medial and Intimal Calcification (A) Despite the extensive presence of MAC, the overlap arc between MAC and IAC is minimal. Representative histologic images (B and E, H&E; C and F, Movat) and the corresponding schematic illustrations (D and G) demonstrating the absence of circumferential overlap or proximity between MAC and IAC. Abbreviations as in Figure 1.

In addition to this quantitative inverse correlation, MAC and IAC were also spatially segregated. The overlap arc was 0° (IQR: 0°–0°) (Figure 7A), indicating that even when both processes were present within the same arterial segment, they did not occur along the same circumferential location. Histological examples further illustrate this absence of circumferential overlap (Figures 7B and 7C). These findings demonstrate that MAC and IAC represent distinct and locally segregated pathological processes within the arterial wall.

## Discussion

In this section-by-section histopathological study of lower extremity arteries in patients with LEAD, we provided a comprehensive characterization of MAC and IAC. By systematically analyzing 3,566 histological sections from 125 arterial segments, we demonstrated that MAC and IAC represent spatially distinct calcific processes with an inverse quantitative relationship. MAC was highly prevalent and substantially more extensive in BTK arteries, often progressing to advanced patterns and bone formation, whereas IAC predominated in ATK arteries. Importantly, the arcs of MAC and IAC were inversely correlated, and circumferential overlap between the 2 was minimal, suggesting that advanced MAC may locally limit the development of intimal atherosclerotic plaque. These findings are summarized in the Central Illustration.

**Central Illustration:**
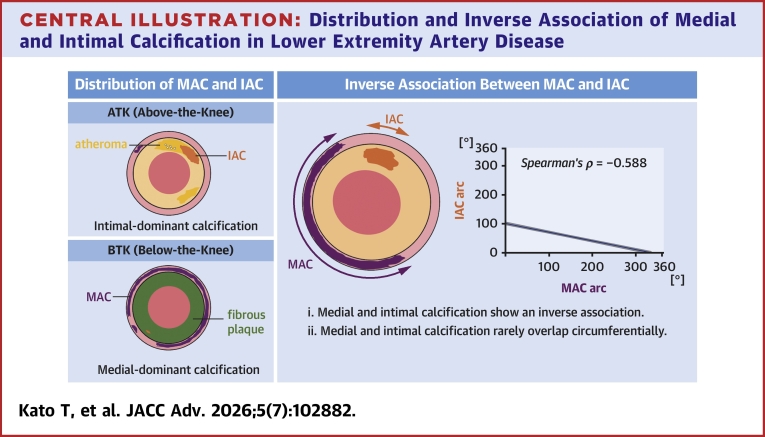
Distribution and Inverse Association of Medial and Intimal Calcification in Lower Extremity Artery Disease ATK arteries predominantly show intimal-dominant calcification associated with atherosclerotic plaques, whereas BTK arteries are characterized by extensive medial-dominant calcification. Quantitative analysis demonstrates an inverse association between MAC and IAC, and spatial analysis reveals minimal circumferential overlap between the 2. ATK = above-the-knee; BTK = below-the-knee; IAC = intimal artery calcification; MAC = medial artery calcification.

### Distribution and extent of medial and intimal calcification

Previous studies have attempted to characterize arterial calcification using radiography, computed tomography, or intravascular imaging. However, these modalities have limited ability to reliably distinguish medial from intimal calcification, particularly when the 2 coexist. For example, Everhart et al^7^ described age- and site-related progression of arterial calcification using plain radiography, but accurate differentiation between MAC and IAC remained challenging. Plain radiography may suggest MAC based on characteristic linear or “railroad track” patterns, whereas intimal calcification tends to appear more focal and irregular. These features are not always specific. Similarly, although intravascular ultrasound and optical frequency domain imaging improve calcium detection, distinguishing medial from intimal deposits remains difficult, particularly when intimal calcium overlies the media.^9^

Pathological investigations have also had important limitations. Soor et al examined more than 1,300 histological slides from amputated limbs but analyzed only the most morphologically abnormal regions, without rigorous plaque classification.^13^ Narula et al^12^ evaluated 239 arterial segments from CLTI patients, yet only 1 section was taken from each artery, limiting the ability to determine true segment-level prevalence and severity of MAC.

In contrast, our study assessed all 3,566 sections from 125 arterial segments, representing a systematic section-by-section histopathological evaluation of lower extremity arteries, with high-resolution assessment of calcification distribution. This comprehensive analysis demonstrated that MAC is overwhelmingly more prevalent and substantially more extensive in BTK than in ATK arteries, with no significant variation among segments within each region.

Importantly, we also identified medial bone formation—a novel and advanced phenotype of MAC—which was strongly associated with the greatest arcs of calcification, suggesting that ossification represents a late stage in the spectrum of MAC progression. Notably, the medial bone formation observed in our study exhibited organized trabecular structures with marrow-like spaces, indicating true ossification rather than amorphous mineral deposition. This finding supports the concept that advanced MAC represents an active, cell-mediated process, rather than a purely passive accumulation of calcium.

Interestingly, although the tibioperoneal trunk is anatomically classified as a BTK artery, its calcification profile more closely resembles that of ATK arteries. Specifically, tibioperoneal trunk segments exhibited a higher prevalence of IAC and a lower burden of MAC compared with the distal tibial arteries. These findings suggest that the tibioperoneal trunk may serve as a transitional zone—both structurally and pathologically—between proximal elastic-predominant arteries and distal muscular arteries. Consequently, the characteristic “BTK pathology,” dominated by extensive MAC, appears to become fully established in the tibial and peroneal arteries distal to the tibioperoneal trunk.

The higher prevalence and severity of MAC in BTK arteries compared with earlier pathological reports likely reflects our high-risk cohort, which included a large proportion of patients with CLTI and hemodialysis. MAC is known to occur predominantly in muscular arteries rich in vascular smooth muscle cells^18^ and to be promoted by aging, diabetes mellitus, CKD, extracellular vesicle–mediated mineral deposition, matrix degradation, metabolic disturbances, and genetic factors.^1^^,^^18^^,^^21^ However, a definitive explanation for the marked concentration of MAC in BTK arteries remains elusive, highlighting the need for further mechanistic studies.

From a clinical perspective, the predominance of MAC, rather than IAC, in BTK arteries may contribute to the suboptimal outcomes observed with established endovascular therapies, such as plain balloon angioplasty.^15^ In the present study, we observed that nonatherosclerotic intima was markedly more prevalent in BTK segments, whereas atherosclerotic intima predominated in ATK arteries, despite no significant difference in the degree of luminal stenosis between anatomical levels. These findings suggest that, particularly in BTK arteries, luminal compromise may arise in the setting of nonatherosclerotic intimal thickening coexisting with extensive medial calcification, rather than being driven primarily by advanced atherosclerotic plaque.

However, extensive MAC may substantially alter vessel wall mechanics by increasing arterial stiffness, reducing compliance, and limiting effective vessel expansion during angioplasty.^1^ In this setting, balloon dilation may lead to vessel wall injury involving deeper layers of the arterial wall rather than effective modification of intimal disease,^22^ which may contribute to limited acute luminal gain and reduced procedural durability.

With the emergence of new endovascular technologies, including drug-eluting resorbable scaffold evaluated in the recent LIFE-BTK trial,^6^ there is increasing interest in lesion-specific therapeutic strategies. Randomized studies have demonstrated the benefit of drug-eluting devices in infrapopliteal arteries, establishing them as an important component of contemporary BTK revascularization strategies; however, the relative performance of different device platforms, including bioresorbable scaffolds vs contemporary drug-eluting stents, remains to be fully established.^23^

In this context, our observation that MAC and IAC are spatially segregated suggests that distinct calcification patterns may differentially influence procedural response. Lesions characterized by predominant intimal disease may be more amenable to therapies targeting plaque modification and vessel scaffolding, whereas segments dominated by extensive MAC may primarily require strategies to address vessel rigidity and limited expandability.

Accordingly, improving outcomes in patients with CLTI may require a more nuanced, lesion-specific approach that considers both intimal pathology and medial calcification, while recognizing that the optimal device selection for different calcification phenotypes remains an area for future investigation.

### Relationship between medial calcification and intimal atherosclerotic plaque

Our findings demonstrate a clear and previously unrecognized dissociation between MAC and intimal atherosclerotic plaque. Sections with extensive MAC tended to exhibit nonatherosclerotic intima, whereas advanced intimal plaque phenotypes—including fibrocalcific plaque and calcified nodules—were more frequently observed in sections with only minimal MAC. Although earlier pathological studies examined the relationship between MAC and lumen stenosis^12^ or between plaque type and stenosis,^11^ and more recent studies have described the distribution of atherosclerosis and intimal thickening in CLTI across the vascular tree,^10^ none clarified the direct interaction between MAC and plaque morphology. Our results indicate that the biological processes driving MAC differ fundamentally from those responsible for atherosclerosis-related IAC, despite their shared systemic risk factors.

A key mechanistic insight centers on the role of vascular smooth muscle cells. Intimal atherogenesis requires migration and proliferation of medial vascular smooth muscle cells, as well as their interaction with macrophage-derived foam cells to form necrotic cores and fibrous caps.^24^, ^25^, ^26^, ^27^ We therefore propose a “medial barrier” hypothesis, in which advanced MAC restricts these processes by entrapping vascular smooth muscle cells within a mineralized matrix or impairing their ability to migrate toward the intima. This concept is strongly supported by our finding that the overlap arc between MAC and IAC was essentially zero, indicating that even when both processes were present within the same arterial segment, they rarely occupied the same circumferential location. Thus, the spatial organization of MAC may actively limit the development of intimal atherosclerosis.

Clinically, these findings suggest that arteries with advanced MAC may be less susceptible to obstructive atherosclerotic remodeling, whereas arteries with minimal MAC may remain vulnerable to plaque progression. Understanding this medial-intimal interplay may refine the interpretation of vascular imaging, improve risk stratification, and inform therapeutic strategies that better target the distinct pathobiology of medial vs intimal disease in patients with LEAD.

### Study limitations

There are several important limitations to this study. First, the number, length, and anatomical distribution of arterial segments varied among subjects, with a predominance of BTK segments, which may have introduced sampling variability and influenced the observed distribution and characteristics of calcification. In addition, the present study did not include foot arteries, such as the dorsalis pedis or plantar arteries, which are increasingly recognized as clinically important in patients with CLTI. Therefore, the applicability of our findings to the most distal vascular territories remains uncertain, and further studies incorporating pedal arteries are warranted to better elucidate the distribution and interaction of medial and intimal calcification in these regions. Second, because the study population consisted largely of patients with CLTI and a high proportion of individuals undergoing hemodialysis, the findings may reflect the pathological characteristics of an advanced, high-risk population and may not be generalizable to patients with earlier stages of LEAD. Nevertheless, this high-risk cohort provides valuable insight into the severe end of the disease spectrum. Third, the study cohort included patients with acute limb ischemia, a condition that may occur in the presence or absence of underlying atherosclerotic disease and may therefore introduce heterogeneity in the pathological substrate. Fourth, although we collected clinical variables relevant to mineral metabolism—including serum phosphorus, calcium, intact parathyroid hormone, and creatinine—these data were incomplete, and in patients on hemodialysis, such parameters may fluctuate substantially depending on the timing of sampling, potentially limiting their interpretability. Fifth, the decalcification process required for histological preparation may have led to partial loss of calcium deposits, especially smaller or more fragile calcifications, resulting in potential underestimation of calcification burden. Sixth, a high proportion of patients had a history of prior revascularization. Although segments with prior stent implantation or surgical grafting were excluded from the analysis, detailed procedural characteristics were not consistently available, and we cannot completely rule out the potential influence of prior interventions on arterial wall structure.

Despite these limitations, this study provides a systematic section-by-section histopathological assessment of MAC and IAC in lower extremity arteries to date and offers important insights into the distinct biology of calcification in LEAD.

## Conclusions

In this comprehensive, section-by-section histopathological study of lower extremity arteries in patients with LEAD, MAC was highly prevalent and markedly more extensive in BTK than in ATK segments, whereas IAC showed the opposite distribution. Advanced MAC patterns and medial bone formation were closely associated with greater circumferential involvement, indicating that ossification represents an advanced stage of MAC. Importantly, MAC was most extensive in sections with nonatherosclerotic intima, and MAC and IAC demonstrated an inverse quantitative relationship with minimal circumferential overlap. These findings suggest that advanced MAC may limit the development of intimal atherosclerotic plaque and may help explain the suboptimal response of BTK lesions to conventional atherosclerosis-targeted therapies, such as balloon angioplasty. A deeper understanding of the medial-intimal interplay may refine imaging interpretation, improve risk stratification, and guide the development of therapies tailored to the distinct biology of LEAD.

Perspectives **COMPETENCY IN MEDICAL KNOWLEDGE:** Medial and intimal calcification represent distinct and spatially segregated processes in lower extremity arteries. Recognition of calcification phenotype may improve interpretation of vascular imaging, which has limited ability to differentiate these entities. A lesion-specific approach considering both medial and intimal pathology may help optimize treatment strategies in LEAD.

**TRANSLATIONAL OUTLOOK:** The mechanisms underlying the spatial segregation and inverse relationship between medial and intimal calcification require further investigation. Improved imaging techniques are needed to enable noninvasive identification of calcification phenotypes. Prospective studies are warranted to determine the impact of calcification phenotype on treatment response and clinical outcomes.

## Funding support and author disclosures

Dr Torii has received research grants from 10.13039/100016242Boston Scientific Japan, 10.13039/100031956Shockwave, and 10.13039/100004374Medtronic; and honoraria from 10.13039/100016242Boston Scientific Japan. Dr Nakazawa serves as a consultant for Boston Scientific, Abbott Vascular, and Terumo Corporation. All other authors have reported that they have no relationships relevant to the contents of this paper to disclose.
